# Bioethics committee approvals in multicentre laryngology research across Europe: the European Laryngological Society’s consensus on legal basis and quality assurance

**DOI:** 10.1007/s00405-026-10057-7

**Published:** 2026-04-29

**Authors:** Malgorzata Wierzbicka, Michal Zabrodsky, Chadwan Al Yaghchi, Christoph Arens, Gauthier Desuter, Necati Enver, Ahmed Geneid, Renata Kopczyk, Isabel Garcia Lopez, Alexia Mattei, Elisabeth Sjögren, Miroslav Tedla, Cesare Piazza

**Affiliations:** 1 Research & Development Centre, Regional Specialist Hospital Wroclaw, Wroclaw, Poland; 2 Faculty of Medicine, Wroclaw University of Science and Technology, Wroclaw, Poland; 3 Department of Otorhinolaryngology and Head and Neck Surgery, First Faculty of Medicine, Charles University, University Hospital Motol, Prague, Czech Republic; 4 National Centre for Airway Reconstruction and the Imperial Voice and Swallowing Centre, Imperial College Healthcare NHS Trust, London, UK; 5 Department of Otorhinolaryngology, Head & Neck Surgery, Justus Liebig University Gießen (JLU Giessen), Gießen, Germany; 6 ENT, Head and Neck Surgery, Antwerp University Hospital, UZA, Edegem, Belgium; 7 Istanbul Voice and Swallowing Center, Istanbul, Turkey; 8 Department of Otolaryngology and Phoniatrics-Head and Neck Surgery, Helsinki University Hospital and University of Helsinki, Helsinki, Finland; 9 Faculty of Management, Wroclaw University of Science and Technology, Wroclaw, Poland; 10 Department of Otolaryngology, La Paz University Hospital, Madrid, Spain; 11 Department of Oto-Rhino- Laryngology and Head and Neck Surgery, APHM, La Conception University Hospital, Marseille, France; 12 Department of Otolaryngology – Head & Neck Surgery, Leiden University Medical Centre (LUMC), Leiden, The Netherlands; 13 Department of Otolaryngology, Head and Neck Surgery, Comenius University, University Hospital, Bratislava, Slovakia; 14 Unit of Otorhinolaryngology–Head and Neck Surgery, ASST–Spedali Civili of Brescia, Brescia, Italy; 15 Department of Medical, Surgical and Radiological Sciences and Public Health, University of Brescia, Brescia, Italy

**Keywords:** Research ethics committees, Multicenter studies, Laryngology research, European regulation, Bioethics oversight, Multinational collaboration

## Abstract

**Purpose:**

To describe European legal foundations, governance structures, and quality assurance mechanisms for bioethics committee approvals in multicenter laryngology research, and present a consensus framework to harmonize practice across Europe.

**Methods:**

An expert panel used the Nominal Group Technique to define scope, review supranational and national regulations (including Regulation (EU) 536/2014 and GDPR considerations), and identify practical barriers in multicenter/multinational laryngology research. Consensus rounds prioritized reproducibility, feasibility, and harmonization of ethics review processes across jurisdictions.

**Results:**

Regulatory context combines EU Regulation 536/2014 and GDPR with substantial national heterogeneity in REC/IRB structure and procedures. Core challenges include: (i) heterogeneity of documentation and approvals; (ii) ambiguity about single vs. multiple REC approvals in multicenter studies; (iii) limited guidance for registry-based and real-world data designs; and (iv) variable integration of data protection and ethics review. Key recommendations: clarify when single-country versus cross-border REC approvals are required; standardize documentation templates and timelines; align consent and data-handling with ethics review; provide governance for emerging study designs and biobanking; and promote EUlevel coordination to harmonize practices.

**Conclusions:**

National variation challenges multicenter laryngology research in Europe. The European Laryngological Society provides a consensus framework to guide legal bases and quality assurance of REC approvals, aiming to improve efficiency, consistency, and ethical compliance.

## Introduction

Contemporary research in the field of laryngology is increasingly characterized by multicenter and multinational collaborations. Such partnerships enable the collection of more extensive datasets, enhance the statistical power of analyses, and facilitate the generalization of findings to diverse patient populations [1, 2]. However, conducting research on this scale presents a range of challenges related to upholding scientific integrity and ensuring compliance with ethical and legal standards [3–5].

The European Laryngological Society (ELS) fosters scientific collaboration within the framework of several topics and projects, engaging researchers from all EU member states as well as associated countries. A central issue is determining which collaborative undertakings require bioethics committee approval, the appropriate scope of such approval, and whether a single collective approval suffices or individual approvals from each institution are necessary [6]. While theoretical studies such as meta-analyses, Delphi, or PRISMA methodologies generally raise fewer ethical concerns, initiatives involving biobanking remain scarce, likely due to uncertainties surrounding transnational permissions.

Bioethics committees, often known as research ethics committees (RECs) or institutional review boards (IRBs), play a pivotal role in the oversight of scientific research involving human participants in Europe. Their primary mandate is to safeguard the rights, safety, and well-being of research subjects, ensuring that studies are conducted in accordance with ethical, legal, and scientific standards [6–9]. For clarity and consistency, the terminology used throughout this manuscript is defined below:

- **Ethics committee** – a general term for a group of experts that reviews ethical issues in clinical care, research, or health policy.
- **Research ethics committee (REC)** – a body that reviews research projects, especially those involving humans or animals, to ensure ethical acceptability.
- **Clinical ethics committee** – an advisory group that helps clinicians and patients resolve difficult ethical dilemmas in everyday clinical practice.
- **Institutional Review Board (IRB)** – the standard term in the United States; a formal committee that reviews, approves, and monitors human research to protect participants’ rights, safety, and welfare.
- **Independent Ethics Committee (IEC)** – a term often used in clinical trials, highlighting that the committee is independent from the sponsor and investigators.
- **Research Ethics Board (REB)** – a term used in several countries (for example, Canada) for a body with the same function as an IRB or REC.
- **Bioethics committee** – a committee that deals with ethical questions in a broader context, such as new technologies, public health policy, or legislation, often advising governments or public institutions.
- **Bioethics board** – a less common term; usually refers to a supervisory or advisory body on bioethics at the level of an institution, region, or state.

The legal framework governing approvals by RECs in Europe is shaped by a multifaceted interplay of supranational regulations, national legislation of Member States, and international soft law instruments. The European legal framework provides a robust foundation for the functioning of these committees. Regulation (EU) No. 536/2014, which superseded Directive 2001/20/EC, is directly applicable in all EU member states and harmonizes the requirements for clinical trials involving medicinal products for human use [10–16]. The regulation requires that clinical trials may only commence after a favorable opinion from REC, which must consider the scientific and ethical validity of the study, the balance of anticipated benefits and risks, the qualifications of investigators, and the adequacy of participant information and consent processes [6, 8, 11]. This regulation introduces a streamlined, single application process and a unified authorization valid across the EU, while granting member states discretion in organizing their ethics committee systems and specifying the details of ethical review [6, 12]. This Regulation also establishes a harmonized authorization procedure through the Clinical Trials Information System (CTIS), mandating that the initiation of any clinical trial be contingent upon a favorable opinion from an ethics committee. It further prescribes specific safeguards for trials involving vulnerable populations, such as minors and incapacitated individuals (Articles 31–35) [15]. Despite this harmonized framework, national laws and administrative practices result in considerable variability in the organization, competencies, and procedures of ethics committees across Europe [6–8, 11].

At the national level, considerable heterogeneity endures [6, 7, 11]. Some jurisdictions adopt centralized regulatory structures characterized by robust normative oversight, exemplified by Lithuania’s Law on Ethics of Biomedical Research (2000), Croatia’s Law on Medicinal Products and Medical Devices (2007), and Slovakia’s Act on Biomedical Research (2005). In contrast, others implement decentralized models, such as the Netherlands’ Medical Research Involving Human Subjects Act (WMO, 1998) and the United Kingdom’s Medicines for Human Use (Clinical Trials) Regulations 2004, administered in tandem with the Health Research Authority’s protocols. This divergence engenders procedural variances in areas including the binding effect of REC opinions, the breadth and rigor of ethical review, requirements for consent documentation, and prescribed administrative timelines.

An additional layer of complexity arises from the interplay with data protection laws, particularly the General Data Protection Regulation (GDPR, Regulation (EU) 2016/679), which governs the processing of personal data in research. GDPR’s Articles 9 and 89 allow for the processing of sensitive health data for scientific purposes under safeguards like pseudonymization or explicit consent but require compatibility with ethical reviews. Discrepancies between research ethics consent (focused on participant autonomy and welfare) and data protection consent (emphasizing lawful processing and rights like erasure) can lead to controversies, especially in cross-border data sharing. International transfers under GDPR pose further challenges for biomedical research, often requiring adequacy decisions or standard contractual clauses.

Recent developments as of 2025 highlight ongoing efforts to address these issues. For instance, the European Federation of Pharmaceutical Industries and Associations (EFPIA) has proposed reinforced coordination between national ethics committees and a single EU-level decision for member states to streamline approvals. Additionally, the MedEthicsEU group, established in February 2024, serves as a forum for national medical research ethics committees to harmonize practices. In specific jurisdictions, such as Denmark, a new 14-day assessment process for Phase I trials was launched in August 2025, setting a benchmark for efficiency. Italy reorganized its ethics committees in February 2025 to align with EU standards.

As a result, the structure, authority, and processes of RECs vary considerably across European countries, influenced by both supranational regulations and national laws. This diversity can present challenges to collaboration in laryngology field, particularly in the context of multicenter, transnational studies and the collection of biological samples for inter-institutional analyses. The objective of this publication is therefore to systematically review European and national regulations governing requirements for REC approvals, and to analyze the legal foundations, competencies, and scope of issues considered by these committees, with the goal of formulating a common position framework applicable to various types of scientific undertakings.

### Method

The Nominal Group Technique (NGT), a robust and formalized method for group consensus-building, was used. NGT combines the creativity of individual brainstorming with the collective wisdom of structured group discussion and prioritization [15, 16]. The rigor, transparency, and inclusivity make it a tool in the clinical guideline’s development, and balanced, democratic decision-making [13, 14, 17]. First step was the election of the expert panel, ensuring representation from various scientific centers and different subspecialties related to the topic (Table 1).

**Table 1:** Key methodological steps

Step	Key actions
Panel formation	International experts’ selection, role assignment, conflict of interest management
Topic and scope definition	Identification of clinical need, explicit framing of questions
Evidence review	Systematic literature search, inclusion of diverse data and different countries perspectives
Consensus process	Formal methodology, iterative feedback, transparent reporting
Drafting recommendations	Synthesis of evidence and expert opinion, clear distinction between evidence and consensus
Review and validation	Internal/external peer review, documentation of contributions
Dissemination	Publication, society communication, implementation guidance

The team precisely defined the thematic scope and objectives of the document, formulating key questions that the recommendations were intended to address, followed by a comprehensive literature review which encompasses both scientific publications and existing guidelines, considering different perspectives, including national values and preferences. Multicenter and international research initiatives introduce additional layers of complexity with respect to ethical governance.

During the NGT rounds, participating experts were asked not only to identify regulatory and ethical issues, but also to articulate recurrent practical barriers encountered in multicenter and multinational research. These barriers were meticulously refined and prioritized across consensus rounds and served as the empirical basis for the final recommendations. The most consistently identified barriers included: (1) heterogeneity of documentation and approval requirements across jurisdictions; (2) uncertainty regarding the acceptability of single versus multiple REC approvals in multicenter studies; (3) lack of guidance for emerging study designs such as registry-based research and real-world data analyses; and (4) inconsistent integration of data protection and ethics review requirements.

Final recommendations were formulated based on the agreed-upon positions and the findings from the literature review. The completed document underwent internal review within the authors group and external peer review, to identify any errors and ensure the highest scientific quality.

This consensus focused on regulatory and ethical governance frameworks; consequently, direct patient or public involvement was not incorporated into the methodology. Nevertheless, the proposed recommendations are intended to strengthen participant protection and transparency, and future ELS initiatives should consider formal integration of patient and public perspectives.

## Results

The analysis of the legal framework governing the activities of RECs in EU member states, in alignment with Regulation (EU) 536/2014, is presented in Table 2. The data were compiled by subject-matter experts and subsequently verified by a legal professional. In 5 out of the 6 examined domains, no significant differences were identified among the 12 countries analyzed.

**Table 2:** Summary of the main legal issues concerning European Regulation (EU 536/2014) and research ethics committees (RECs) in the member states (MS)

Principle	EU	France	Italy	Belgium	Germany	Netherlands	Finland
Committee definition	Independent, multidisciplinary	39 independent and multidisciplinary committees distributed across the territory	Decentralized, multicenter, multidisciplinary committees	Decentralized to universities and universities affiliated institutions NL: Ethisch Comité FR: Comité d’éthique hospital-facultaire	Decentralized, university- or medical association-based, multidisciplinary committees (‘’Ethikkommission’’)	24 accredited Regional and a Central Committee on Research Involving Human Subjects (CCMO), which oversees studies of high complexity	Decentralized to universities and universities affiliated institutions
Scope of review	Clinical trials on medicinal products	All research involving human participants	All biomedical research	All biomedical research	All biomedical research	All biomedical research	All biomedical research
Application process	Single EU-wide application	Random allocation of a committee after submission on a national dedicated platform	Single application via the Technical-Scientific Secretariat	Institutions standardized SOP	Institutions standardized SOP	Single, although the CCMO supervises the RECs	Institution standardized
Approval requirement	Mandatory before research starts	Mandatory before research starts	Mandatory before research starts	Mandatory before study starts Amendments possible	Mandatory before study begin Amendments possible	Mandatory before study begin	Mandatory before research
Membership requirements	Medical and non-medical members	Medical and non-medical members	Medical and non-medical members	MD and non-MDs	Medical and non-medical members	Multidisciplinary experts	Medical members
Additional safeguards	For vulnerable populations	Opinion on the exemption from the obligation to inform the person who provided a biological sample following a change in the purpose of a sample collection/a sample from the human body	Special attention to vulnerable populations, informed consent, data protection, use of biological samples	According to: 1/Royal decreet 12/08/1994 2/ EU directive 2011/20 3/ Belgian laws of 7/5/2004 and 19/03/2012 on human experimentations Federal medication agency decreet 613	According to: German Medicines Act (§ 40–42 AMG), German Medical Devices Act (§ 31–38 MPDG) and General Data Protection Regulation (DSGVO)	Netherlands Code of Conduct for Research Integrity; (1/9/ 2018) Code of Conduct defines five principles of research integrity, and 61 standards Code has been adopted by Royal Academy of Arts and Sciences (KNAW), Federation of University Medical Centers (NFU), Organization for Scientific Research (NWO), Associated Applied Research Institutes (TO2 federation), Association of Universities of Applied Sciences and Association of Universities in the Netherlands (VSNU)	For vulnerable populations

Principle	Slovakia	Poland	Spain	Czech Republic	UK	Turkey
Committee definition	Decentralized, governed by the Health Care Act, and operate within the Ministry of Health, universities, and hospitals	Decentralized, regional committees. Founded at universities and regional medical healthcare associations	Decentralized, hospital committees. multidisciplinary, independent	Decentralized hospital and institutional committees, multidisciplinary, independent, in compliance with EU ethical standards (e.g., Regulation (EU) No. 536/2014) and Czech national law	Independent, regional, multidisciplinary committees established by the Health Research Authority (HRA)	Independent, university/ training and research hospital/ provincial health directorate-based, multidisciplinary committees
Scope of review	All biomedical research	All biomedical research	Investigation projects on human beings or biological material	All ethical, legal, and scientific aspects related to the protection of human research participants	All health and social care research involving human participants, their data, or tissue	Separate RECs for clinical trials on medicinal products and other biomedical research
Application process	Single EU	Single	Single	Single, Institutional standardized process	Centralized through the Integrated Research Application System (IRAS)	Institution’s standardized SOP
Approval requirement	Mandatory before research starts	Mandatory before research starts, with possible additional steps/modifications while the research is ongoing	Mandatory before research starts	Mandatory before research starts	Mandatory before research begins; substantial amendments also require approval	Mandatory before research starts Amendments possible
Membership requirements	EC evaluate the qualifications of a proposed research team, having the authority to reject the entire study if their expertise is deemed insufficient	Medical members	Medical and non-medical members (for instance: nurse, investigators, pharmacologist, lawyer, patient representatives	Medical and non-medical members	Both medical and non-medical backgrounds; lay and expert members included	Medical and non-medical members
Additional safeguards	Comprehensive informed consent, robust data protection, specific protections for vulnerable populations, and ongoing oversight to ensure participant safety and ethical compliance throughout a clinical trial	National rules are more specific	NO, they follow what is called standard operating procedures	Yes. Multiple safeguards such as independent, multidisciplinary ethics committees, dual review by SÚKL and ERCs, mandatory insurance, and strict data-protection rules to ensure participant safety and transparency Additional protections apply to vulnerable groups, requiring enhanced consent procedures and oversight to guarantee that participation is voluntary and ethically justified	Specific provisions for vulnerable groups (e.g., children, those with impaired consent capacity); aligned with UK regulations and the Mental Capacity Act	Special consent and expert involvement for vulnerable populations (children, pregnant and lactating women, incapacitated individuals)

Table 3 provides a detailed characterization of the institutional structures, operational procedures, and functional scope of local RECs across Europe. For each country, the allocation of responsibilities regarding informed consent, data protection, and the management of complications, adverse events, and incidental findings were clearly delineated, based on the best available expert knowledge. Notable differences between countries were observed in the domains of reporting the source of funding and confirmation of principal investigator insurance. In contrast, consensus was virtually unanimous across all jurisdictions with respect to key elements such as the study protocol and the patients’ written informed consent form.

**Table 3:** Required documentation and characteristics for research bioethics committee review forms across countries

Document	France	Italy	Belgium	Germany	Netherlands	Finland	Slovakia	Poland	Spain	Czech Republic	UK	Turkey
Research protocol: title, institutional affiliation, applicant, principal investigator, coordinator, research team, study duration and rationale, recruitment strategies	++	++	+	++	++	++	++	++	++	++	++	++
Specification of intended study population in relation to objectives and endpoints	++	++	+	++	++	++	++	++	++	++	++	++
Inclusion and exclusion criteria with scientific, medical, and ethical justification	++	++	+	++	++	++	++	++	++	++	++	++
Protocol detailing relevant demographic and non-demographic variables	++	++	+	++	++	++	++	++	++	++	++	++
Accessibility considerations for participants with disabilities (physical accommodations, transportation, translation)	-	+	+	-	++	++	+	-	-	++	+	-
Report of total number of enrolled participants by demographic and non-demographic characteristics	- (not at the beginning)	++	+	++	++	+	+	++	++	++	+	++
Report of withdrawn participants by demographic and non-demographic characteristics	- (not at the beginning)	+	+	+	++	-	+	+	+	+	+	+
Participant-facing materials: study information for patients (plain language, numeracy, design, visualization)	++	++	+	++	++	++	++	++	++	++	++	++
Patients’ written informed consent form	++	++	+	++	++	++	++	++	++	++	++	++
Study funding statement indicating source(s) of funding	++	++	+	++	++	++	++	++	++	++	++	++
Rationale for absence of participant compensation (if applicable)	+	Data non available	+	+	+	++	+	+	-	++	+	+
Principal investigator insurance confirmation	The insurance subscriber is generally the promoter	++		++	All participants of clinical trials are insured	All participants of clinical trials are insured as part of their participation	++	++	-	++	++	++ (only for trials without a commercial sponsor, for the participants)
Institutional insurance confirmation	++	++	+So called “No fault Insurance” covering all clinical investigators validated by the REC	++	++	All participants of clinical trials are insured as part of their participation	++	++	-	+	++	++ (from the commercial sponsor, for the participants)
Statement on distribution of potential research benefits	-	+	-	+	++	+	++	+	+	+	+	++
Availability of REC resources: protocol templates, guidelines	yes	yes	yes	yes	yes	Yes	Yes	yes	Yes	yes	Yes	yes
Availability of REC resources: training, and educational tools for investigators/sponsors	yes	yes	Valid GCP certificates mandatory for all investigators	Valid GCP certificates mandatory	yes	yes	No	no	No	yes	Valid GCP certificates mandatory for all investigators	Valid GCP certificates mandatory
How many types of studies/applications for approval offer RCB? *	5	>5	>7		>5	>5	>5	>5	>5	>5	>7	≥6
How many forms need to be completed within a single application? **	≥9	>5	>7		>5	>5	>5	>5	>5	3	>5	≥12
What is the range (minimum–maximum) of fees charged by the RCB for processing an application (in Euro)?	none	Fixed fees, data not available	none	0-5000		None	0-1200	100-2000	0	0-1000	none (publicly funded studies); commercial: £500–£6000+	0-1500
Do individual RCB differ in terms of the level of detail required in applications and the fees charged for their review?	No individual RCB for research involving human participants	yes	none	yes	yes	yes	yes	yes	yes	yes	yes	none

Legend:

Not required, + Recommended, ++ Strictly required,

* Exemplarily: Non-commercial clinical trial, Commercial clinical trial, Doctoral thesis, Postdoctoral thesis, Research using a non-standard method/drug intervention, Research experiment in the field of basic sciences, Decision on exemption from having an RCB Opinion,

** Exemplarily: Research protocol, Patients’ written informed consent form, Study funding statement, Institutional insurance confirmation, Principal investigator insurance confirmation, others

Classical categories of scientific studies—specifically, traditional research involving human subjects, their data, or biological materials, which unequivocally require prior REC approval are summarized in Table 4. In these aspects, complete concordance was observed among the countries under review. The second part of Table 4 enumerates emerging registry-based research (reg-search) and unprecedented study designs, which have not yet been standardized in terms of ethical oversight and therefore warrant careful consideration. Finally, the Recommendations of the European Laryngological Society (ELS) on the Role of Research Ethics Committees (RECs) in Multicenter and Multinational Research are presented in Table 5.

**Table 4:** Types of classical scientific studies and emerging registry-based research requiring ethics committee approval, with particular emphasis on multicenter and international collaboration

Type of study	France	Italy	Belgium	Germany	Netherlands	Finland	Slovakia	Poland	Spain	Czech Republic	UK	Turkey
Preclinical studies utilizing human biological material (e.g., tissues, cells, biobanking initiatives)	+	++	+	++	++	++	++	++	++	++	++	++
Medical experiments involving human participants (including first-in-human applications of novel diagnostic or therapeutic strategies)	++	++	+	++	++	++	++	++	++	++	++	++
Non-commercial clinical trials (such as academic, investigator-initiated studies)	++	++	+	++	++	++	++	++	++	++	++	++
Commercial clinical trials (industry-sponsored interventional studies)	++	++	+	++	++	++	++	++	++	++	++	++
Doctoral dissertations and post-doctoral theses involving original research with human subjects or their data/materials	++	++	+	++	++	++	++	++	++	++	++	++
Case reports and case series containing identifiable patient information or requiring consent for publication	+	++	+	++	++	++	+	++	++	++	++	++
Meta-analyses and systematic reviews PRISMA methodology	+	-	+	+	-	++	-	-	++	-	-	-
Meta-analyses and systematic reviews that entail primary analysis of unpublished or non-anonymized data	++	Data non available	+	++	+	++	+	+	++	++	+	++
research that involves the integration or linkage of multiple registries or databases,	+	++	+	++	++	++	+	+	++	++	+	++
cross-border data sharing, particularly within the framework of international or multicenter collaborations	+		+	++	++	++	+	+	++	++	+	++
Delphi designed studies	+	+	+	++	+	++	-	-	-	++conditional	-	+
A single RCB application and approval is sufficient for multicenter studies.	Yes (approval on a national platform)	+ (if Clinical Trials Information System procedure is adopted, but regional regulation does not explicitly state single application for all centers	No each participating institutions are asked to validate the study by their own local REC	No One main application and validation in further local study centers	REC or Central Committee (CCMO) needed but not always sufficient. Each participating institution must also provide a local "Site Suitability Declaration" (Verklaring Geschiktheid Onderzoeksinstelling, VG)	+	+	+	+	+	Yes – via IRAS for UK-wide REC approval	+(preferably obtained from the REC of coordinating center or from one of the RECs in the same province)
Separate RCB applications and approvals are required for each participating center in multicenter studies.	no	Regional regulation does not explicitly refer to the need of separate approval	yes	yes	yes	no	-	-	-(It is required to have one of them approved in Spain	yes	-	-

- Not required, + Recommended, ++ Strictly required,

The European Research Council Ethics requirements in your ERC application. Carolina C. Ávila 6 March 2025 https://erc.europa.eu/sites/default/files/2025-03/Guidance_on_Research_Ethics_requirements.pdf  1.Human Embryonic Stem Cells and Human Embryos 2.Human participants 3.Human cells / tissues 4. Personal data 5. Animals 6. Non-EU countries 7. Environment & Health and Safety 8. Artificial Intelligence 9. Other ethics issues

**Table 5:** Recommendations of the European Laryngological Society (ELS) on the role of research ethics committees (RECs) in multicenter and multinational research

1. Recognition of Emerging Study Designs RECs should extend their oversight to novel methodological frameworks such as registry-based research, large-scale real-world data analyses, and unprecedented multicenter study designs, which present unique risks of re-identification and cross-border data governance challenges.
2. **Adaptive Models of Ethical Review** To address the heterogeneity of national requirements, the ELS recommends piloting adaptive review mechanisms that combine centralized approval for ethical principles with targeted local feasibility reviews, thereby avoiding duplication while respecting jurisdiction-specific legal contexts.
3. **Integration of Data Protection Safeguards Beyond GDPR Minimums** In light of mounting complexity in health data linkage, REC approval processes should explicitly validate enhanced safeguards—including dynamic consent models and governance structures for secondary use—that go beyond GDPR baseline compliance, to ensure robust participant trust in international research collaborations.
4. **Tailored Ethical Oversight According to Study Typology** The ELS proposes differentiated REC engagement tailored to the research category: ○ For meta-analyses and systematic reviews (e.g., PRISMA), REC oversight should focus on transparency in data sourcing, secondary use permissions, and privacy safeguards for unpublished datasets. ○ For consensus-building methodologies (e.g., Delphi), emphasis should be placed on protecting participant confidentiality, informed consent for expert contributions, and safeguarding intellectual independence. ○ For primary patient data collection or biobanking, RECs should rigorously assess protocol integrity, the robustness of consent procedures (including broad consent for future use), and cross-border material/data transfer governance.
5. **Priority for Early-Stage REC Consultation in Collaborative Consortia** Multicenter laryngology studies, particularly those involving biological materials or transnational registries, should engage RECs at the earliest planning stage to facilitate regulatory alignment of approvals, pre-empt conflicts between ethical and data protection requirements, and expedite study initiation within multinational frameworks.

Importantly, although substantial agreement was observed across countries regarding core ethical requirements, the expert panel consistently identified operational and procedural barriers that were not fully captured by existing regulations. These shared barriers—particularly those related to approval pathways in multicenter studies, emerging research designs, and cross-border data governance—directly informed the formulation of the ELS recommendations presented in Table 5.

## Discussion

This paper presents an analysis of the legal framework governing the activities of RECs in EU member states, in line with Regulation (EU) 536/2014, alongside a characterization of the institutional structures, operational methods, and functional scope of local RECs. The structure, authority, and procedures of RECs vary widely across Europe, reflecting both supranational frameworks and national legislation. This diversity poses barriers to collaborative laryngology research, particularly in multicenter, cross-border studies and in the exchange of biological materials. Reviewing European and national regulations governing REC requirements—including their legal foundations, competencies, and scope—was essential to advancing a unified position applicable across different categories of scientific projects [18–20].

While EU regulations set minimum standards, limitations exist in common law. Member states retain significant discretion in organizing RECs, the binding nature of their opinions, and their interactions with regulatory authorities [11]. Consequently, a patchwork of systems has emerged, posing challenges for multinational studies, especially those not involving medicinal products, where procedural consistency is less developed [21]. An additional knowledge gap concerns emerging scientific challenges, such as reg-search and unprecedented study designs [22, 23].

To the best of our knowledge, this is the first collaborative initiative among clinicians in the field of laryngology that seeks to compare the diversity of approaches and identify potential inconsistencies, with the overarching aim of advancing the protection of research participants. The development of a scientific society, ELS consensus statement by a panel of experts ensures the credibility and authority of the final part of this document.

### National variations in bioethics committee approvals

National approaches to REC approval vary widely, reflecting differences in regulatory philosophy, administrative structure, and cultural context. Some countries, such as Croatia, Lithuania, and Slovakia, have centralized systems with strict oversight [8, 11], while the majority—including the Netherlands, Poland, Italy, Spain, France, Belgium, Finland, the Czech Republic, Turkey, and the United Kingdom—adopt decentralized models. Procedural differences include the scope of review, consent requirements, and administrative timelines. These differences also underscore the critical importance of securing local ethical approval, not only to ensure compliance with national legislation, but also to guarantee that research is conducted in a manner sensitive to the cultural, social, and regulatory context of the host country [11, 21]. International guidelines, including the Declaration of Helsinki, emphasize the importance of local committee approval to protect participant rights and foster equitable research partnerships [11, 21]. EU regulations do not consistently clarify whether the opinions of RECs are legally binding, nor do they provide a precise delineation of the respective responsibilities assigned to RECs. However, our analysis demonstrates that additional safeguards, as well as a firm anchoring within national legislation, are clearly articulated.

Procedural divergences are nevertheless evident. For example, while the United Kingdom mandates formal ethical review for virtually all research involving human participants, the Netherlands exempts certain non-interventional studies from compulsory REC oversight. Further disparities arise in requirements for consent and data governance: in some jurisdictions, verbal consent is deemed sufficient for interview-based studies, whereas others stipulate written documentation. Notably, the scope of review is often defined in overly general terms, with most countries applying the broad formulation “all biomedical research.” By contrast, approval requirements are strikingly uniform: across the 12 jurisdictions examined, there is consensus that failure to secure appropriate local approval jeopardizes both the ethical integrity of a study and community trust [24], particularly in research conducted across borders or involving vulnerable populations [25].

In light of these complexities, it is imperative that researchers engage in careful advance planning, taking into account national differences in ethical review procedures when designing and conducting multinational studies [11, 21, 26].

### Emerging reg-search and unprecedented study designs in multicenter analyses

In recent years, there has been a marked increase in reg-search, novel study designs leveraging large-scale health data repositories, electronic health records, and real-world data. Our analysis demonstrates that in such projects securing approval from a REC is strongly recommended, particularly when the research involves the integration or linkage of multiple registries or databases, which may elevate the risk of subject re-identification. REC approval is also advisable in cases of cross-border data sharing, especially within the framework of international or multicenter collaborations, a scenario frequently encountered in the activities of scientific societies.

A distinct category comprises systematic reviews conducted in accordance with PRISMA methodology, which traditionally, do not mandate formal ethical approval, as they synthesize and analyze data from previously published studies [27–29]. However, taking into consideration large and diverse consortia, if a systematic review entails the collection of unpublished data from individual study authors, or incorporate data not already available in the public domain, ethical approval may be required.

The necessity for ethical approval from REC in Delphi studies largely depends on whether the project qualifies as “research involving human subjects” and on the nature of the data collected. The need for approval often hinges on whether the project is classified as research, audit, or service evaluation [30, 31]. Especially in health or biomedical research, obtaining ethical approval is considered best practice [30, 32–34]. However, there is still some debate and inconsistency in the field regarding when ethical approval is strictly required [34].

For biobanking or the banking of biological material [35], RECs should mandate adherence to best practices in informed consent, material transfer agreements, governance, and international standards, thereby safeguarding both the ethical and legal dimensions of cross-border research cooperation [35–37].

A single REC or IRB application and approval is increasingly recognized as sufficient for multicenter studies in many jurisdictions, particularly in North America and Europe [38–42]. This approach, often referred to as “single IRB review” or “centralized ethical review”, aims to streamline the ethical approval process, reduce administrative redundancy, and facilitate the timely initiation of multicenter research. For instance, the NIH requires a single IRB for all its multicenter studies, with participating sites accepting the central approval while retaining responsibility for local feasibility and compliance. Similarly, the UK’s Health Research Authority mandates a single REC review for multicenter research, restricting local site review to operational considerations rather than duplicating ethical assessment [43–46]. However, it is important to note that while a single REC/IRB approval is generally sufficient from an ethical standpoint, each participating center typically must conduct a local feasibility assessment and provide administrative authorization before research can commence at that site. This local review does not duplicate the ethical assessment but ensures that the study can be safely and effectively conducted given the local context, resources, and population.

Emerging designs must also navigate GDPR intersections, where ethical approvals should confirm safeguards for health data processing, including broad consent for future uses under Article 89. New EU regulations on shared ethics reviews, effective from January 2025, aim to improve efficiency in real-world data (RWD) studies by allowing centralized assessments for registries.

As a result of the ensuing debate, prompted by significant discrepancies among national approaches at the outset of the joint work, a set of common final statements was developed. Here are three core consensus statement principles, synthesizing novel elements relevant to contemporary ELS research ethics practice and multicenter collaboration: (1) Innovation-Responsive Oversight. Research ethics committees should be structurally equipped to address and proactively review emerging scientific designs—including registry-based research and large-scale real-world data projects—ensuring that ethical protections and data governance standards evolve with methodological innovation. (2) Adaptive and Differentiated Ethical Review. REC processes should be tailored to the specific typology of collaborative research, with distinct attention to meta-analyses, consensus-building methodologies, and primary patient data protocols. This includes nuanced application of data privacy safeguards and customized consent requirements consistent with the nature of participant involvement and dataset sensitivity. (3) Integrated, Early-Stage Ethics Consultation. All multicenter collaborations should initiate REC engagement at the earliest research planning phase—prioritizing coordination of approvals, pre-emptive resolution of regulatory complexity, and alignment of procedural standards. Early ethics integration fosters robust international collaboration and mitigates cross-jurisdictional barriers.

Within multicenter laryngology collaboration, both classical and emerging types of scientific research involving human subjects, their data, or biological materials should be subjected to rigorous ethical review. This is particularly critical in the context of international collaborations, where the complexity of legal and ethical requirements is significantly heightened. It is strongly recommended that investigators seek guidance from the appropriate ethics committees at the earliest stage of project development to ensure full compliance with all applicable ethical standards and regulations.

To summarize, the legal landscape governing RECs within the EU is characterized by a complex interplay between national legislation of member states and supranational regulation, which, while mandating the involvement of independent ethics committees in the scientific and ethical review of clinical trials, leaves considerable discretion to individual member states regarding the scope, binding nature, and procedural details of REC assessments [6, 11, 47]. However, still significant national differences persist in the structure, authority, and processes of these committees, lasting for over 20 years [48–51].

### Limitations

This study has some limitations regarding patient and public involvement (PPI). Patient and public involvement is increasingly recognized as a hallmark of high-quality, ethical health research, as it entails working collaboratively with patients and members of the public to shape research questions, study design and dissemination strategies, rather than conducting research merely *to* or *about* them. In the present study, which focuses on the regulatory architecture and formal procedures of RECs, no formal PPI activities (such as patient advisory panels or co-designed workshops) were undertaken, because the work relied on the analysis of policies, guidelines and legal documents rather than on the collection of data from patients or the public. Nonetheless, given that RECs play a central role in protecting research participants, future empirical research on REC practice should incorporate structured PPI, for example by involving patients and public contributors in the evaluation of application processes, participant information materials and the communication of REC decisions [52, 53].

## Conclusion

Bioethics committee approvals are a cornerstone of ethical research in Europe, mandated by both international guidelines and European legislation. While EU regulations have fostered some degree of consistency, many challenges remain, particularly for non-drug research and multinational studies. Ongoing efforts to clarify roles, standardize procedures, and promote mutual recognition of ethical reviews are essential for advancing ethically sound and scientifically robust research across Europe. In the context of scientific collaboration within the ELS, it is recommended that the engagement of RECs be tailored to the nature of the collaborative endeavor: for meta-analyses and systematic reviews (e.g., PRISMA), RECs should ensure compliance with data privacy and secondary use regulations; for consensus-building methodologies (such as those adopting the Delphi modality), ethical oversight should focus on participant confidentiality and informed consent; for primary patient data collection, RECs must rigorously assess protocol design, consent procedures, and data protection measures.
